# Biological soliton in multicellular movement

**DOI:** 10.1038/srep02272

**Published:** 2013-07-29

**Authors:** Hidekazu Kuwayama, Shuji Ishida

**Affiliations:** 1 Faculty of Life and Environmental Sciences, University of Tsukuba, Tsukuba, Tennodai, 1-1-1, Ibaraki 305-8572, Japan; 2 Department of Botany, Graduate School of Science, Kyoto University, Sakyo-ku, Kyoto 606-8502, Japan

## Abstract

Solitons have been observed in various physical phenomena. Here, we show that the distinct characteristics of solitons are present in the mass cell movement of non-chemotactic mutants of the cellular slime mould *Dictyostelium discoideum*. During starvation, *D. discoideum* forms multicellular structures that differentiate into spore or stalk cells and, eventually, a fruiting body. Non-chemotactic mutant cells do not form multicellular structures; however, they do undergo mass cell movement in the form of a pulsatile soliton-like structure (SLS). We also found that SLS induction is mediated by adhesive cell-cell interactions. These observations provide novel insights into the mechanisms of biological solitons in multicellular movement.

Solitons are a widely observed physical phenomenon that behave like waves but possess many features of particles^1^. A soliton is defined as a self-reinforcing solitary wave that travels at constant speed without changing shape. Solitons do not obey the superposition principle, which makes the wave structure robust in collisions with other wave structures. In biology, soliton theory has been applied to explain signal and energy propagation in biomembranes, the nervous system, and low frequency collective motion in proteins and DNA^2^,^3^,^4^,^5^; however, there has been no evidence of solitons as a higher-level biological phenomena. An example of such biological phenomena is multicellular movement during morphogenesis and development. The details of how this organized phenomenon is spatiotemporally regulated have not been reported, but presumably such regulation is characterized by simple and robust rules.

We have demonstrated that non-chemotactic *Dictyostelium discoideum* mutants exhibit a characteristic structure with the features of a self-reinforcing solitary wave, or soliton. Under starvation conditions, the mutants do not aggregate but form an arc-shape multicellular structure, named the Soliton-Like-Structure (SLS). SLS movement continues much longer than the developmental cycle, moves at constant speed without changing shape, and does not obey the superposition principle. Even after collisions, waves pass through each other, conserving their physical qualities. Thus, we conclude the SLS exhibits soliton features that may be maintained by cell-to-cell adhesion mechanisms.

## Results

### Non-chemotactic mutants of *Dictyostelium discoideum* exhibit a soliton-like structure in multicellular movement

**Figure 1:**
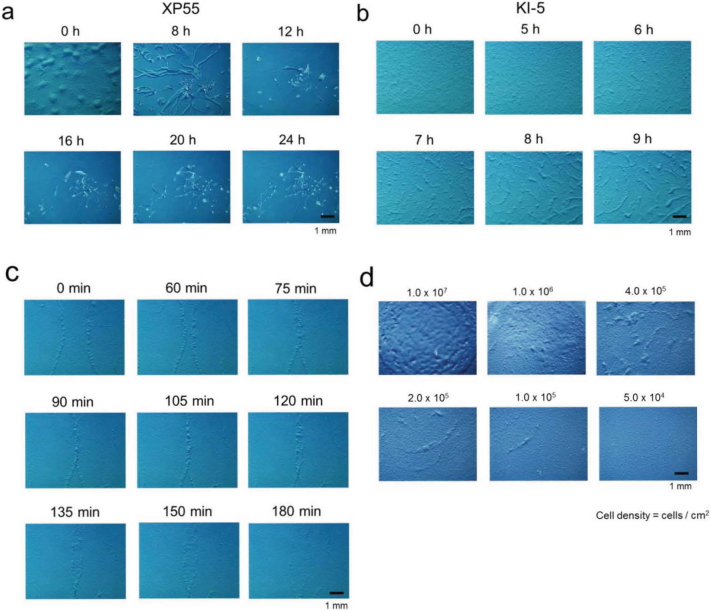
Multicellular movement of the non-chemotactic *D. discoideum* KI-5 mutant shows soliton-like structures (SLSs) which behave similarly to soliton waves. (a, b), Formation of multicellular structures in parental wild-type XP55 (a) and SLSs in non-chemotactic mutant KI-5 (b) cells. (c), The collision of 2 independent SLSs. (d), Dose dependency of KI-5 cell density on SLS formation. All pictures were taken at the indicated time after exhaustion of the *D. discoideum* food source, *Klebsiella aerogenes*.

In wild-type cells of the slime mould *Dictyostelium discoideum*, a starvation signal triggers a developmental programme. Following exhaustion of their bacterial food source, the erstwhile random movement of the amoeboid cells is orchestrated to yield an aggregation of cells, mediated by the self-secreted extracellular chemoattractant, cAMP. The resultant migratory, multicellular structure is known as a slug. Transformation of the slug occurs within 8 h and results in a fruiting body that consists of two differentiated cell types, spore cells and stalk cells^6^ (Fig. 1a, Supplementary video S1).

We have isolated *D. discoideum* mutants that lack all chemotactic activities, leaving them unable to proceed down developmental pathways that require cell aggregation^7^,^8^. Soliton-like structures (SLSs) were observed in the KI-5 and KI-10 mutants within 6 h following the consumption of bacteria (Fig. 1b, Supplementary video S2). Since the features of the SLSs were not distinguishable between the two mutants, we only describe the KI-5 phenotype in this manuscript. SLSs emerged around 6 h after exhaustion of the bacterial food supply and persisted for 48 ± 3 h in 5 independent experiments. (See a typical time course of SLS in Supplementary Fig. S1 and Supplementary video S3). Maintenance of the SLS structure despite collision with other SLSs is characteristic of a soliton wave (Fig. 1c, Supplementary video S4). The formation of SLSs is dependent on cell density such that the higher the density of the cells, the larger the SLS formed (Fig. 1d); however, an excessively high density prevents SLS formation (Fig. 1d). Moreover, as the duration of cell starvation increased, SLS size and number decreased (Supplementary Fig. S1, Supplementary video S3). Despite the variability in size, SLSs moved at a constant velocity of approximately 20 μm/min, which is twice the rate (10 μm/min) of starved wild-type cells^9^,^10^. Since SLS velocity is comparable to that of prespore or prestalk cells in the slug^11^, SLS cell motility may represent a distinct aspect of differentiated cell movement.

### SLS depends on the cAMP signalling genes but is independent of extracellular cAMP and DIF-1

**Figure 2:**
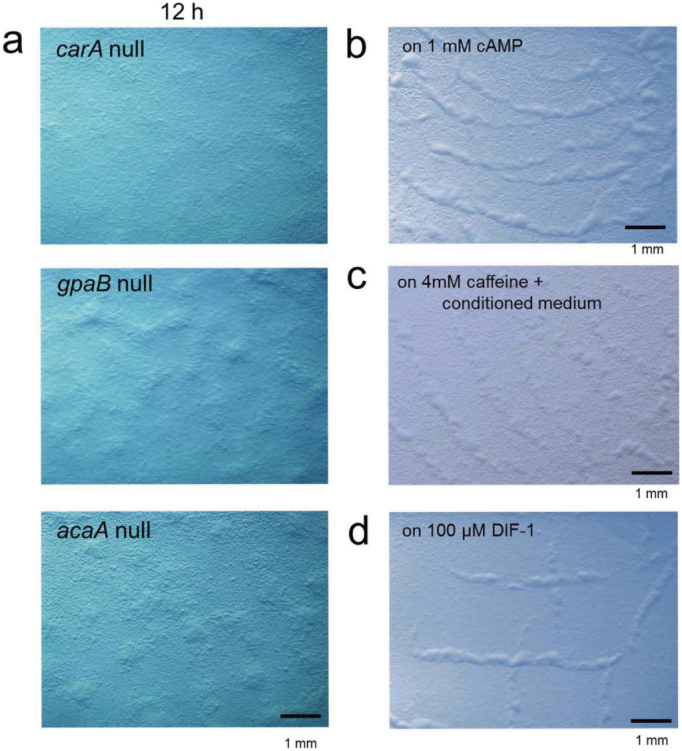
Failure of SLS formation in non-chemotactic mutants with defective cAMP signalling, and the effect of cAMP and DIF-1 on SLS formation. (a), Absence of multicellular structure formation in *carA*, *gbpB*, and *acaA* null mutants following bacterial exhaustion. (b), SLS formation with 1 mM cAMP. (c), Absence of SLS formation with 4 mM caffeine. (d), SLS formation with 100 μM DIF-1.

In *D. discoideum*, the sensory signalling pathway used during development involves cAMP synthesis and signalling through the serpentine cell surface receptor, trimeric G protein^12^. Therefore, mutants that lack the genes responsible for these processes—*carA*; the cAMP receptor, *gpaB*; the trimeric G protein α subunit, and *acaA*; an adenylyl cyclase—are defective in cell aggregation and dependent processes due to lack of cAMP production^13^,^14^,^15^. These null mutants showed few signs of SLS formation, indicating that the cAMP signalling pathway is required for SLS formation (Fig. 2a, Supplementary video S5, S6, S7). We have shown that KI-5 and KI-10 are defective in cGMP metabolism in response to extracellular cAMP stimuli despite possessing the machinery for cAMP receptor-dependent cAMP production^7^,^16^,^17^. During cell aggregation, extracellular cAMP concentrations of an order of magnitude less than the micromolar level play a critical role such that cAMP concentrations of a millimolar order of magnitude substantially inhibit morphogenesis^18^. Next, in order to determine if extracellular cAMP is required for SLS formation, KI-5 cells were exposed to extremely high concentration and depletion of cAMP. Under the condition with the high concentration of 1 mM cAMP, formation of SLSs was not disturbed (Fig. 2b). Furthermore, KI-5 cells were plated on non-nutrient agar with 4 mM caffeine and conditioned medium prepared from the supernatant of wild-type cells in starvation (see Methods). Since caffeine is an adenylyl cyclase inhibitor in *D. discoideum* and the conditioned medium contains enough phosphodiesterase activity to degrade most of the physiological amount of extracellular cAMP^19^,^20^, little cAMP was measured in the extracellular fraction of KI-5 cells starved for 12 h with 5 mM caffeine and conditioned medium (Table I; see Supplementary methods). With 5 mM caffeine and the conditioned medium, SLSs were normally formed without disturbance (Fig. 2c). These observations indicate that extracellular cAMP is not required for SLS formation. Another morphogen, DIF-1, is a stalk cell-inducing factor and a modulator of chemotactic activity^21^,^22^. In the presence of high DIF-1 concentrations, SLSs developed normally (Fig. 2d).

### SLS is a dynamically stabilized structure

**Figure 3:**
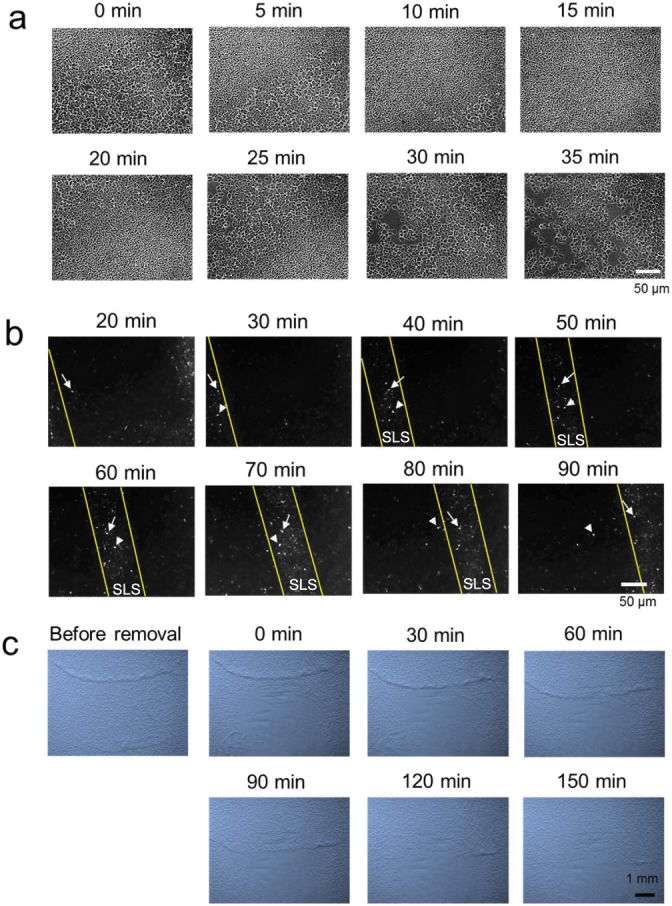
Cell movements in SLSs. (a), Cell movement of SLSs. (b), Cell movement of SLSs with RFP-labelled KI-5 cells. (c), Removal of cells in front of a moving SLS. The arrow and arrowhead indicate cells incorporated into and left behind an SLS, respectively.

Cell motion in SLSs was investigated at a higher magnification and with RFP (Red fluorescent protein)-expressing KI-5 cells generated by transformation with monomeric RFP expression vector. Motile cells in front of the wave moved randomly and did not appear to migrate into the SLS but instead were incorporated passively (Fig. 3a, 3b & Supplementary video S8, S9, S10). At the same time, some cells at the rear of the SLS were left behind and reverted to random motion. The number of RFP-expressing incorporated cells was comparable to the number of cells left behind the SLS (Fig. 3b). In 3 independent SLSs, the number of highly RFP-expressing incorporated cells and the number of cells left behind were nearly the same (Table II). Furthermore, removal of cells in front of a moving SLS showed that the thickness of the SLS was reduced and eventually disappeared (Fig. 3c, Supplementary video S11). These results indicate that SLSs do not constitute a static, independent cluster of cells but are dynamically stabilized structures that continuously incorporate cells in front and leave cells trailing behind.

### Collision of two independent SLSs

**Figure 4:**
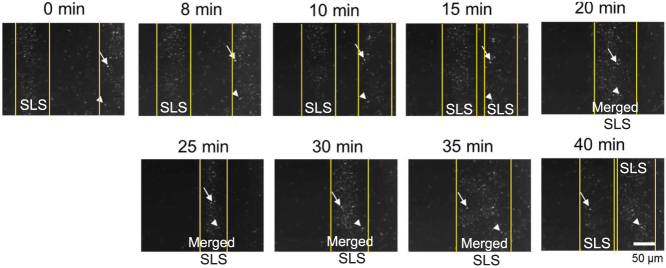
Collision of two independent SLSs with RFP-expressing KI-5 cells. The arrow and arrowhead indicate cells which maintained and changed direction, respectively, in SLSs after collision.

The SLS structure is stable regardless of collision other SLSs (Fig. 1c, Supplementary video S4). In order to investigate cell behaviour in colliding SLSs, SLS collisions were recorded at high magnification. In head-on collisions, the SLSs appeared to coalesce as clusters and glide in a circular motion (Supplementary video S11). They did not always maintain their original orientation, with some switching orientation to the opposite direction. This was confirmed in RFP-expressing KI-5 cells (Fig. 4, Supplementary video 12, 13). Some cells in each soliton maintained (arrow in Fig. 4) or reversed (arrowhead in Fig. 4) direction after the collision. These results indicate that, in SLS collisions, cells merge into each other as a cluster and glide, but separate in 2 directions independent of the original direction.

### End-to-end adhesion may play a major role in the distinct and stable directional locomotion of SLS

**Figure 5:**
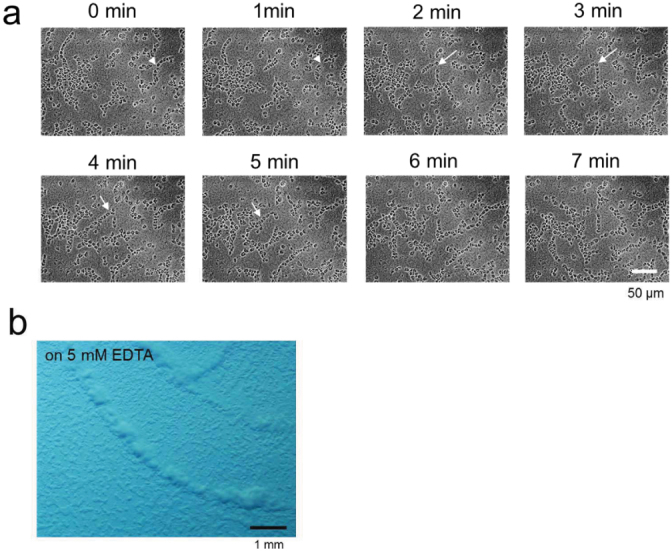
Movement of the dispersed cells derived from SLSs. (a), Cell movement of loosely disaggregated SLS. Arrows indicate breakage of a cell concatenate. Arrowheads indicate attachment of 2 cell concatenates. (b), SLS formation on an agar plate containing 5 mM EDTA.

Next, cells comprising SLS were moderately dispersed and placed under an agar overlay (Fig. 5a, Supplementary video 14). A population of cells contacted each other in an end-to-end manner and moved in a tandem structure, similar to cell movement in slugs^23^. Within this structure, two or more cells were often found to concatenate although they sometimes formed a broken line (arrows in Fig. 5a). These end-to-end concatenations and subsequent tandem structure movements were observed on occasions when one cell encountered other cells (arrowheads in Fig. 5a). Furthermore, SLS formation was never inhibited by 5 mM EDTA at which concentration calcium-dependent cell adhesion is abolished and, thus, cell aggregation in wild-type cells is blocked by inhibition of side-by-side cell adhesion^24^ (Fig. 5b). These observations suggest end-to-end adhesion may play a major role in the distinct and stable directional locomotion of SLS, and calcium-dependent cell adhesion is not involved in SLS formation.

## Discussion

In this manuscript, we show that the distinct characteristics of solitons are observable in the mass cell movement of non-chemotactic mutants of a cellular slime mould. Upon starvation, wild-type cells of the cellular slime mould *D. discoideum* stop growing and initiate spontaneous formation of multicellular structures, accompanied by chemotaxis-dependent cell aggregation. This results in cell differentiation. In contrast, non-chemotactic mutants do not undergo aggregation; however, they do undergo a mass cell movement that possesses the characteristics of a soliton. They form pulsatile SLSs that retain their shape and move at a constant velocity. Furthermore, the shape of each SLS remains unchanged after collisions with other SLSs (Supplementary video S4, S12, S13). These observations provide the first insight into soliton-related behaviours in eukaryotic multicellular movement.

In myxobacteria, starved cells exhibit a highly organized periodic pattern of accumulations that move as traveling waves called ripples^25^,^26^. Two sets of waves appear to move in opposite directions with the same wave length and speed, like SLSs; however, the mechanism of wave movement in collisions is rather different from that of SLS. When two ripple wave fronts collide, cells move in the reverse direction. Consequently, the outgoing waves consist of a combination of individuals from both incoming waves. This gives the appearance of waves passing through one another. In the collision of SLSs, each cell maintains its direction forward or reverses. This feature is distinctly different from that of SLS.

Null mutants that lack *carA*, *gpaB*, and *acaA* are defective in SLS formation. These observations indicate that cAMP signalling is responsible for SLS formation. On the other hand, extracellular cAMP and exhaustion of cAMP by caffeine had no effect on SLS formation. Thus, cAMP signalling is important for the transition from vegetative to starved stage, but is not necessary for SLS maintenance. Furthermore, since KI-5 is defective in intracellular cGMP metabolism in response to cAMP stimuli, cGMP is not necessary for SLS emergence and maintenance.

In *D. discoideum*, cell adhesion molecules mediate cell-to-cell contact during development. DdCAD-1 and gp80 are responsible for spatio-temporal cell adhesion. DdCAD-1 localizes primarily on cells at the tip and outer margin of cell streams, whereas gp80 localizes to contact regions between cells within well-developed streams^27^,^28^. DdCAD-1 is enriched in filopodial structures at the cell contact region, suggesting DdCAD-1 plays an important role at the leading edge of migrating cells. However, since we found that SLS formation and maintenance are EDTA-resistant phenomena, the adhesion molecule is not likely to be responsible for SLS, because DdCAD-1 is not adhesive under calcium-depleted conditions in the presence of EDTA. In the end-to-end adhesion in SLS, calcium-resistant adhesion molecules may serve as a target marker for contact. Based on these observations, the explanation for dynamically stable movement of SLS may be elucidated through the specific localization of calcium-resistant adhesive molecules such as gp80.

Based on our observations, we speculate that the formation of SLSs is initiated by random collisions in early-stage starvation. Since SLS is only observed under starvation conditions, we suggest cell polarization is important for SLS. Once cells become polarized, their movement becomes aligned and propagates as a cluster by end-to-end adhesion. To understand if the characteristics of biological solitons reported in this study can apply to multicellular movement in not only *D. discoideum* but also general developmental morphogenesis, further mathematical treatment providing theoretical validation of the soliton nature of the SLSs is required.

## Methods

### Culture conditions and SLS observation

The parental strain of wild-type *Dictyostelium discoideum* XP55 and the non-chemotactic mutants KI-5 and KI-10 cells were cultivated as described^7^. For each mutant, 1.0 × 10^5^ cells were inoculated on a 9-cm 1/3 SM plate (0.3% glucose, 0.3% bactopeptone, 1.5% agar, and 40 mM KH_2_PO4/Na_2_HPO_4_, pH 6.0) with an aliquot of Klebsiella aerogenes suspension. At 2 days incubation at 21°C, bacterial exhaustion was observed. Time-lapse videos were then taken at 21°C with a digital stereoscopic microscope (Olympus SZX12). Conditioned medium was prepared from the extracellular supernatant of wild-type cells (1.0 × 10^7^ cells/mL) incubated in phosphate buffer (PB: 10 mM Na/K phosphate buffer, pH 6.5) for 12 h at 21°C. To look at the effect of cell density on SLS, cells were cultivated on 1/3 SM plates at 21°C. They were harvested and washed 3 times with PB using a repeated centrifugation procedure (350 g for 2 min × 3 at 4°C). The cells of each strain were plated with 1.5% non-nutrient agar and were adjusted to the cell density indicated in the figure legend. Higher-magnification time-lapse videos were taken using a modified agar overlay method as described^29^. Pictures and videos were taken using a contrast and fluorescence microscope (Zeiss Axiovert 200M).

### Transformation of KI-5 cells

Transformation of KI-5 cells was performed as described previously with a few modifications^30^. Details are provided in the Supplementary methods.

## Author Contributions

H.K. performed all the experiments. H.K. and S.I. contributed equally to the discovery of the SLS. H.K. drafted and contributed to the final manuscript.

## Supplementary Material

Supplementary Information Supplementary video S1

Supplementary Information Supplementary video S2

Supplementary Information Supplementary video S3

Supplementary Information Supplementary video S4

Supplementary Information Supplementary video S5

Supplementary Information Supplementary video S6

Supplementary Information Supplementary video S7

Supplementary Information Supplementary video S8

Supplementary Information Supplementary video S9

Supplementary Information Supplementary video S10

Supplementary Information Supplementary video S11

Supplementary Information Supplementary video S12

Supplementary Information Supplementary video S13

Supplementary Information Supplementary video S14

Supplementary Information Supplementary Information

## Figures and Tables

**Table 1:** cAMP concentration in the extracellular fraction of KI-5 cells with 5 mM caffeine and conditioned medium

Addition	cAMP concentration in supernatant (pmol/mL)
No caffeine and no CM	1.70 ± 0.33
5 mM caffeine and CM	0.09 ± 0.06

The results are shown as the mean ± S.D. of triplicate determinations of 3 independent experiments. CM = conditioned medium (see Materials and methods).

**Table 2:** The number of highly REP-expressing cells that are incorporated into and left behind a SLS

	The number of RFP-expressing		
	incorporated cells	cells behind	ratio
Experiment I	17	17	1.0
Experiment II	17	20	0.8
Experiment III	18	20	0.9

Ratio indicates the relative value of the number of RFP-expressing incorporated cells to cells behind a SLS per movie.

## References

[b1] Lakshmanan M. Solitons, tsunamis and oceanographical applications of. in Extreme Environmental Events (ed Meyers, R. A.) 873–888 Springer, (2011).

[b2] Hasegawa A. An historical review of application of optical solitons for high speed communications. Chaos 10, 475–485 (2000). 12779400 10.1063/1.1286914

[b3] Heimburg T. & Jackson A. D. On soliton propagation in biomembranes and nerves. Proc. Natl. Acad. Sci. USA 102, 9790–9795 (2005). 15994235 10.1073/pnas.0503823102 PMC1175000

[b4] Sinkala Z. Soliton/exciton transport in proteins. J. Theor. Biol. 241, 919–927 (2006). 16516929 10.1016/j.jtbi.2006.01.028

[b5] Yakushevich L. V. Is DNA a nonlinear dynamical system where solitary conformational waves are possible? J. Biosci. 26, 305–313 (2001). 11568475 10.1007/BF02703739

[b6] Williams J. G. *Dictyostelium* finds new roles to model. Genetics 185, 717–726 (2010). 20660652 10.1534/genetics.110.119297 PMC2907197

[b7] Kuwayama H., Ishida S. & Van Haastert P. J. Non-chemotactic *Dictyostelium discoideum* mutants with altered cGMP signal transduction. J. Cell Biol. 123, 1453–1462 (1993). 7902839 10.1083/jcb.123.6.1453 PMC2290906

[b8] Ohtsuka H. Relationship between the movements of slug cells and chemotaxis in *Dictyostelium discoideum*. Master's Thesis., Kyoto University, Japan (in Japanese). (1994).

[b9] Pakes N. K. *et al.* The Rac GEF ZizB regulates development, cell motility and cytokinesis in *Dictyostelium*. J. Cell Sci. 125, 2457–2465 (2012). 22366457 10.1242/jcs.100966

[b10] Sato M. J. *et al.* Switching direction in electric-signal-induced cell migration by cyclic guanosine monophosphate and phosphatidylinositol signaling. Proc. Natl. Acad. Sci. USA 106, 6667–6672 (2009). 19346484 10.1073/pnas.0809974106 PMC2672521

[b11] Siegert F. & Weijer C. J. Three-dimensional scroll waves organize *Dictyostelium* slugs. Proc. Natl. Acad. Sci. USA 89, 6433–6437 (1992). 1631140 10.1073/pnas.89.14.6433 PMC49515

[b12] Swaney K. F., Huang C. H. & Devreotes P. N. Eukaryotic chemotaxis: a network of signaling pathways controls motility, directional sensing, and polarity. Annu. Rev. Biophys 39, 265–289 (2010). 20192768 10.1146/annurev.biophys.093008.131228 PMC4364543

[b13] Sun T. J. & Devreotes P. N. Gene targeting of the aggregation stage cAMP receptor cAR1 in *Dictyostelium*. Genes Dev. 5, 572–582 (1991). 1849108 10.1101/gad.5.4.572

[b14] Kumagai A. *et al.* Regulation and function of G alpha protein subunits in *Dictyostelium*. Cell 57, 265–275 (1989). 2539262 10.1016/0092-8674(89)90964-1

[b15] Pitt G. S. *et al.* Structurally distinct and stage-specific adenylyl cyclase genes play different roles in *Dictyostelium* development. Cell 69, 305–315 (1992). 1348970 10.1016/0092-8674(92)90411-5

[b16] Kuwayama H., Viel G. T., Ishida S. & Van Haastert P. J. Aberrant cGMP-binding activity in non-chemotactic *Dictyostelium discoideum* mutants. Biochim. Biophys. Acta 1268, 214–220 (1995). 7662711 10.1016/0167-4889(95)00082-4

[b17] Kuwayama H. & Van Haastert P. J. Regulation of guanylyl cyclase by a cGMP-binding protein during chemotaxis in *Dictyostelium discoideum*. J. Biol. Chem. 271, 23718–23724 (1996). 8798595 10.1074/jbc.271.39.23718

[b18] Van Haastert P. J., Jastorff B., Pinas J. E. & Konijn T. M. Analogs of cyclic AMP as chemoattractants and inhibitors of *Dictyostelium* chemotaxis. J. Bacteriol. 149, 99–105 (1982). 6274850 10.1128/jb.149.1.99-105.1982 PMC216596

[b19] Alvarez-Curto E., Weening K. E. & Schaap P. Pharmacological profiling of the *Dictyostelium* adenylate cyclases ACA, ACB and ACG. Biochem. J. 401, 309–316 (2007). 16952277 10.1042/BJ20060880 PMC1698679

[b20] Shapiro R., Franke J., Luna E. J. & Richard H. Kessin. A comparison of the membrane-bound and extracellular cyclic AMP phosphodiesterases of *Dictyostelium discoideum*. Biochim. Biophys. Acta 758, 49–57 (1983).

[b21] Kay R. R., Flatman P. & Thompson C. R. DIF signalling and cell fate. Semin. Cell Dev. Biol. 10, 577–585 (1999). 10706822 10.1006/scdb.1999.0341

[b22] Kuwayama H. & Kubohara Y. Differentiation-inducing factor-1 and -2 function also as modulators for *Dictyostelium* chemotaxis. PLoS One 4, e6658 (2009). 19684855 10.1371/journal.pone.0006658 PMC2722026

[b23] Umeda T. & Inouye K. Possible role of contact following in the generation of coherent motion of *Dictyostelium* cells. J. Theor. Biol. 219, 301–308 (2002). 12419659 10.1006/jtbi.2002.3124

[b24] Müller K. & Gerisch G. A specific glycoprotein as the target site of adhesion blocking Fab in aggregating *Dictyostelium* cells. Nature 274, 445–459 (1978). 566857 10.1038/274445a0

[b25] Welch R. & Kaiser D. Cell behavior in traveling wave patterns of myxobacteria. Proc Natl. Acad. Sci. USA 98, 14907–10912 (2001). 11752438 10.1073/pnas.261574598 PMC64957

[b26] Igoshin O. A. & Oster G. Rippling of myxobacteria. Math. Biosci. 188, 221–233 (2004). 14766104 10.1016/j.mbs.2003.04.001

[b27] Sesaki H. & Siu C. H. Novel redistribution of the Ca^2+^-dependent cell adhesion molecule DdCAD-1 during development of *Dictyostelium discoideum*. Dev. Biol. 177, 504–516 (1996). 8806827 10.1006/dbio.1996.0181

[b28] Harloff C., Gerisch G. & Noegel A. A. Selective elimination of the contact site A protein of *Dictyostelium discoideum* by gene disruption. Genes Dev. 3, 2011–2019 (1989). 2515990 10.1101/gad.3.12a.2011

[b29] Fukui Y., Yumura S. & Yumura T. K. Agar-overlay immunofluorescence: high-resolution studies of cytoskeletal components and their changes during chemotaxis. Methods Cell Biol. 28, 347–356 (1987). 3298995 10.1016/s0091-679x(08)61655-6

[b30] Kuwayama H. & Nagasaki A. Desalted deep sea water increases transformation and homologous recombination efficiencies in *Dictyostelium discoideum*. J. Mol. Microbiol. Biotechnol. 14, 157–162 (2008). 17693704 10.1159/000107371

