# Supplementary material for: Biological soliton in multicellular movement
Source: Sci Rep. 2013 Jul 29;3:2272. doi: 10.1038/srep02272 (PMC3725511; doi:10.1038/srep02272)
Supplement: Supplementary Information [file srep02272-s15.pdf]

Supplementary Information

Biological Soliton in Multicellular Movement

Hidekazu Kuwayama & Shuji Ishida

Supplementary METHODS

Transformation of KI-5 cells

Transformation of KI-5 cells was performed as described with a few modifications, because KI-5 is not an axenic strain and can only grow with bacteria as its food source[33]. Briefly, KI-5 cells grown on a 1/3 SM agar plate with an aliquot of *Klebsiella aerogenes* were harvested and resuspended in electroporation buffer (10 mM $NaPO_4$, 50 mM sucrose, pH 6.1) with 10 μg of an mRFP expression vector, pHK12neo-mRFP at a density of 5×10^7 cells. A 400-μl aliquot of the cell suspension in a 2-mm gapped cuvette was electroporated at 500 V, 100 μs, 10 times with 1 s intervals. Following electroporation, the cell suspension was incubated at 21°C for 15 min with 4 μl of 100 mM $CaCl_2$ and $MgCl_2$, and then 20 mL HL5 was added to the electroporated cell suspension. The dish containing the electroporated cells was incubated for 24 h at 21°C followed by the addition of 20 μg/mL G418 (Sigma, MO, USA). Ten days later, following careful removal of the floating dead cells, cells were inoculated on 1/3 SM agar with an aliquot of *Klebsiella aerogenes* suspension for several days. Fluorescence and the SLS-related phenotype of one of the isolated clones were examined and used as RFP-expressing KI-5 cells.

Measurement of Extracellular cAMP

To measure extracellular cAMP in KI-5, cells at a stage of SLS formation were harvested and resuspended in 10 mM phosphate buffer, pH. 6.5 for 12 h at a density of 1×10^7 cells/mL with or without 5 mM caffeine and conditioned medium. After shaking for 30 min at 125 rpm at 21°C and centrifugation, the supernatant was harvested for measurement of extracellular cAMP. The cAMP concentration was measured as described[7].

Supplementary Video legends

Supplementary video S1. **Formation of the multicellular structure of parental wild-type XP55 after exhaustion of the bacterial food source**. The time-lapse video was recorded over 24 h and compressed to 28 sec.

Supplementary video S2. **Formation of SLS in the non-chemotactic mutant, KI-5 after exhaustion of the bacterial food source**. The time-lapse video was recorded over 9 h and compressed to 36 sec.

Supplementary video S3. **Formation of SLS in the non-chemotactic KI-5 mutant after exhaustion of the bacterial food source**. The time-lapse video was recorded over 54 h and compressed to 36 sec.

Supplementary video S4. **The collision of two KI-5 SLSs.** The time-lapse video was recorded over 180 min and compressed to 18 sec.

Supplementary video S5-S7. **Absence of multicellular structure formation by *car*A (4), *gbp*B (5), and *aca*A (6) null mutants after exhaustion of the bacterial food source.** The time-lapse video was recorded over 36 or 24 h and compressed to 36 sec (5, 6) or 24 sec (7).

Supplementary video S8. **Cell movement in SLS under an agar overlay at high magnification.** The time-lapse video recorded over 60 min and compressed to 72 sec.

Supplementary video S9-S10. **Cell movement in SLS with RFP-expressing KI-5.** The time-lapse video was recorded on phase-contrast (12) and fluorescence microscopes (13) over 3 h and compressed to 15 sec.

Supplementary video S11. **Removal of cells in front of a moving SLS.** The time-lapse video was recorded over 2.5 h and compressed to 15 sec.

Supplementary video S12-S13. **Cell movement in colliding SLSs of RFP-expressing KI-5.** The time-lapse video was recorded on phase-contrast (12) and fluorescence microscopes (13) over 75 min and compressed to 6 sec.

Supplementary video S14. **Cell movement of cells dispersed from SLS.** The time-lapse video was recorded over 1 h and compressed to 72 sec.

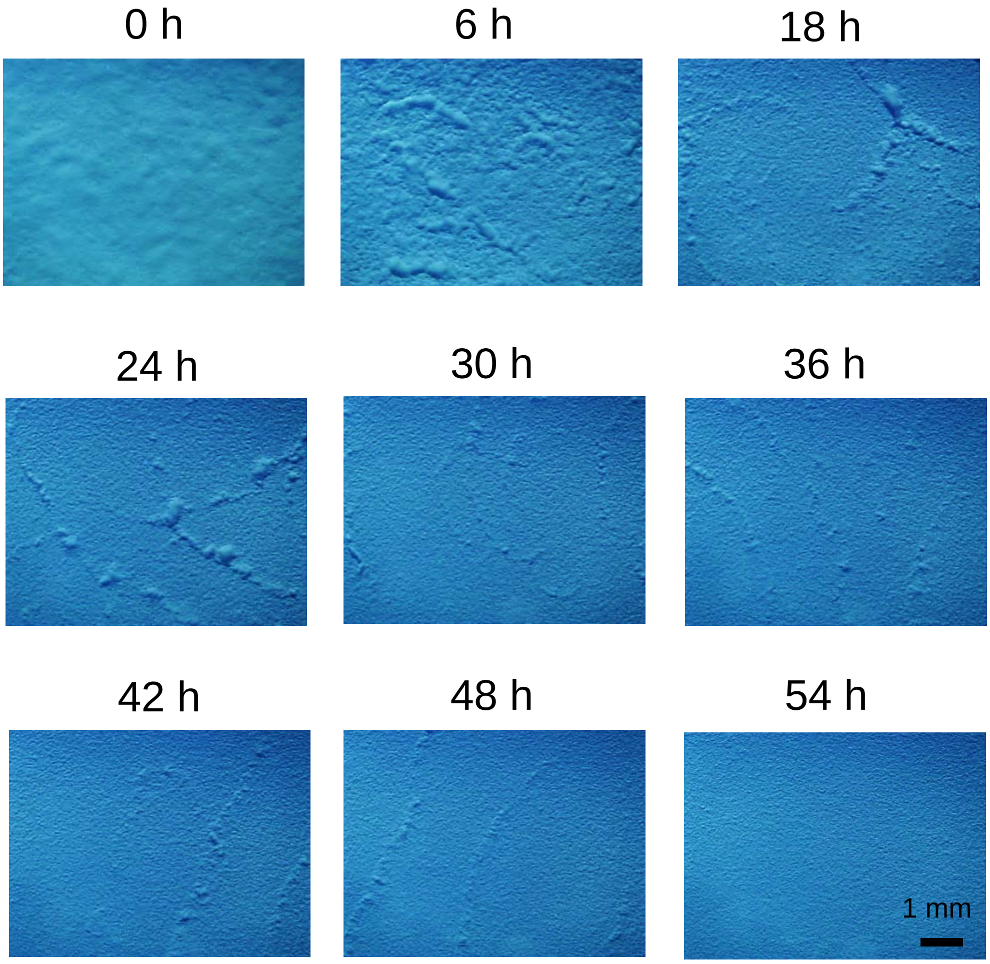

Figure S1 **Formation and time course of SLS in KI-5**
